# Anesthetic dilemmas in an achondroplastic patient undergoing elective cesarean section

**DOI:** 10.7555/JBR.37.20230301

**Published:** 2024-06-04

**Authors:** Aaron Brown, Hong Liu, Cristina Chandler

**Affiliations:** Department of Anesthesiology and Pain Medicine, University of California Davis Health, Sacramento, CA 95817, USA

**Keywords:** achondroplasia, skeletal dysplasia, combined spinal-epidural anesthesia, cesarean delivery

## Abstract

Achondroplasia is a genetic condition characterized by skeletal dysplasia that results in characteristic craniofacial and spinal abnormalities. It is the most common form of short-limbed skeletal dysplasia. A morbidly obese pregnant patient warrants specific anatomical and physiological considerations, such as a difficult airway with potential hypoxia, full stomach precautions, and a reduced functional residual capacity. Achondroplasia increases the risks of maternal and fetal complications. Although neuraxial techniques are generally preferred for cesarean sections, there is no consensus among patients with achondroplasia. We aimed to discuss the anesthetic challenges in an achondroplastic patient and report our regional anesthesia approach for an elective cesarean section.

## Introduction

Achondroplasia is the most common type of disproportionate dwarfism (approximately 1 in 30000 births) caused by an abnormal function of a protein, namely fibroblast growth factor receptor (FGFR). The result is a slowdown of bone growth at the growth plate, causing characteristic craniofacial and spinal abnormalities: macrocephaly with limited neck extension, midfacial retrusion, large tongue, severe kyphosis, and scoliosis^[1‒2]^. Foramen magnum stenosis, atlantoaxial instability, and large mandible and tongue may lead to potentially difficult airway for intubation. Patients with restrictive lung disease and decreased functional residual capacity, as well as variable spine anatomy with spinal cord stenosis and unpredictable spread of local anesthetic, may present a challenge for anesthesia providers^[2‒5]^. For a cesarean section (C-section), neuraxial techniques are generally preferred^[6]^; however, in patients with achondroplasia, there is no unanimity in the choice, anesthetic dose, and management of anesthetic techniques^[7]^. In the current case report, we discuss the anesthetic challenges in an achondroplastic patient undergoing an elective C-section.

## Case report

The patient, a 33-year-old woman (gravida 2 para 1) with morbid obesity and achondroplasia, was scheduled for a repeat elective C-section at 37 weeks and five days. She had a height of 122 cm and a weight of 74.6 kg (body mass index: 50.1 kg/m^2^). Her previous surgical history included a foramen magnum decompression and stabilization surgery at the age of eight months, as well as a C-section. Her first C-section was likely performed under regional anesthesia, although the history was unclear. Her past medical history was significant for obstructive sleep apnea, although she was currently not on continuous positive airway pressure. She had a prior tonsillectomy and adenoidectomy as a child and also had a history of scoliosis, polycystic ovary syndrome, iron deficiency anemia on replacement therapy, and gastroesophageal reflux. Her current pregnancy was otherwise uncomplicated, but there was a concern for cephalopelvic disproportion, thus the obstetric team and patient opted for an early elective C-section delivery. During the preoperative physical exam, the patient appeared well but displayed specific achondroplastic features including short limbs, macrocephaly with a prominent forehead, short neck, large tongue, and scoliosis with prominent lordosis. No prior anesthetic details or spine imaging were available. The airway exam revealed a good mouth opening with a Mallampati class Ⅰ view, but a limited neck range of motion. Significant laboratory values included a hemoglobin level of 9.1 g/dL, a platelet count of 276 thousand per cubic millimeter, and a negative result for the point of care SARS-CoV-2 antigen test. After discussing all anesthetic options and associated risks, the patient consented to regional anesthesia.

Preoperatively, an 18-gauge intravenous (IV) catheter was established, a lactated Ringers infusion was started, 10 mg of metoclopramide was administered intravenously, and 30 mL of sodium citrate/citric acid (Bicitra, [500 mg-334 mg]/5 mL) was administered orally. The patient was positioned in a sitting position in the operating room. In addition to the standard American Society of Anesthesiologists monitors (electrocardiogram, pulse, non-invasive blood pressure [BP], and peripheral oxygen saturation), an arterial line was placed in the left radial artery. The patient's BP was 134/82 mmHg. The back skin was disinfected with chlorhexidine and 4 mL of 1% lidocaine was used for local anesthesia. A combined spinal epidural (CSE) procedure was performed at the L4–L5 intervertebral space using a standard needle-in-needle technique. Loss of resistance to saline was observed at 4 cm from the skin. Using a 25-gauge spinal needle, 10 μg of fentanyl, 100 μg of morphine, and 6 mg of hyperbaric bupivacaine 0.75% were injected intrathecally, for a total of 1 mL. An epidural catheter was left in place at a depth of 9 cm from the skin. The patient was positioned in a supine position with a 15-degree left tilt after urinary catheterization. A phenylephrine infusion was started at 0.5 μg·kg^−1^·min^−1^ to prevent supine hypotension. Fourteen minutes after the spinal injection, no sensory or motor block was achieved (the sensory block was checked with a bilateral pinprick test). Consequently, the epidural catheter was titrated starting with 3 mL of 2% lidocaine with 1∶100 000 epinephrine. Additional 2 mL epidural boluses of the local anesthetic solution were administered at 6, 11, and 17 min, for a total of 9 mL of 2% lidocaine. An appropriate surgical block to the T5 level was achieved 21 min after the initial epidural bolus. BP remained stable throughout. Seven minutes after the onset of surgery, the baby was delivered with APGAR (appearance, pulse, grimace, activity, and respiration) scores of 8 and 9 at the 1^st^ and 5^th^ min marks, respectively. After umbilical cord clamping, an oxytocin bolus of 3 IU was followed by an infusion at 41.7 milliunits per minute. The patient expressed some discomfort as the uterus was exteriorized, so 2 mg of midazolam and 50 mg of fentanyl were IV administered, and the patient responded well. The surgical block was rechecked and remained at the T5 level. The uterine tone was adequate, and no significant hemorrhage was observed. At the end of the 58-min procedure, the phenylephrine infusion was stopped, and 1 g of acetaminophen, 4 mg of ondansetron, and 30 mg of ketorolac were IV administered. The patient remained hemodynamically stable with spontaneous breathing *via* a face mask throughout the procedure. The patient was taken to the recovery room, and the recovery period was complicated by postoperative nausea, which was successfully treated with 12.5 mg of promethazine intravenously. The BP was stable, and no vasopressor support was required. The sensory block decreased by two dermatomes, and the motor function returned after approximately 2.5 h. The epidural catheter was removed before leaving the recovery room. A 24-h evaluation showed a complete return of the patient's sensory and motor functions to the pre-procedure level.

## Discussion

Anesthesia management in achondroplastic patients is challenging, but successful anesthesia has been achieved for C-sections in such cases. The methods included general, single-shot spinal, epidural, and CSE anesthesia^[8]^. Each method presents unique difficulties in gravid achondroplastic patients, and the choice of anesthetic technique needs to be tailored to each patient and their clinical situations. We chose a regional technique instead of general anesthesia (GA) because of the potential risks associated with a difficult airway, failed intubation, and aspiration. Additionally, regional anesthesia offers better postoperative outcomes in terms of pain control and recovery time, as well as improved maternal satisfaction. We opted for a CSE technique considering the repeat cesarean delivery in a morbidly obese patient with a potentially prolonged procedure time and the presence of spinal abnormalities with unpredictable spread of local anesthetic agents.

**Figure 1:**
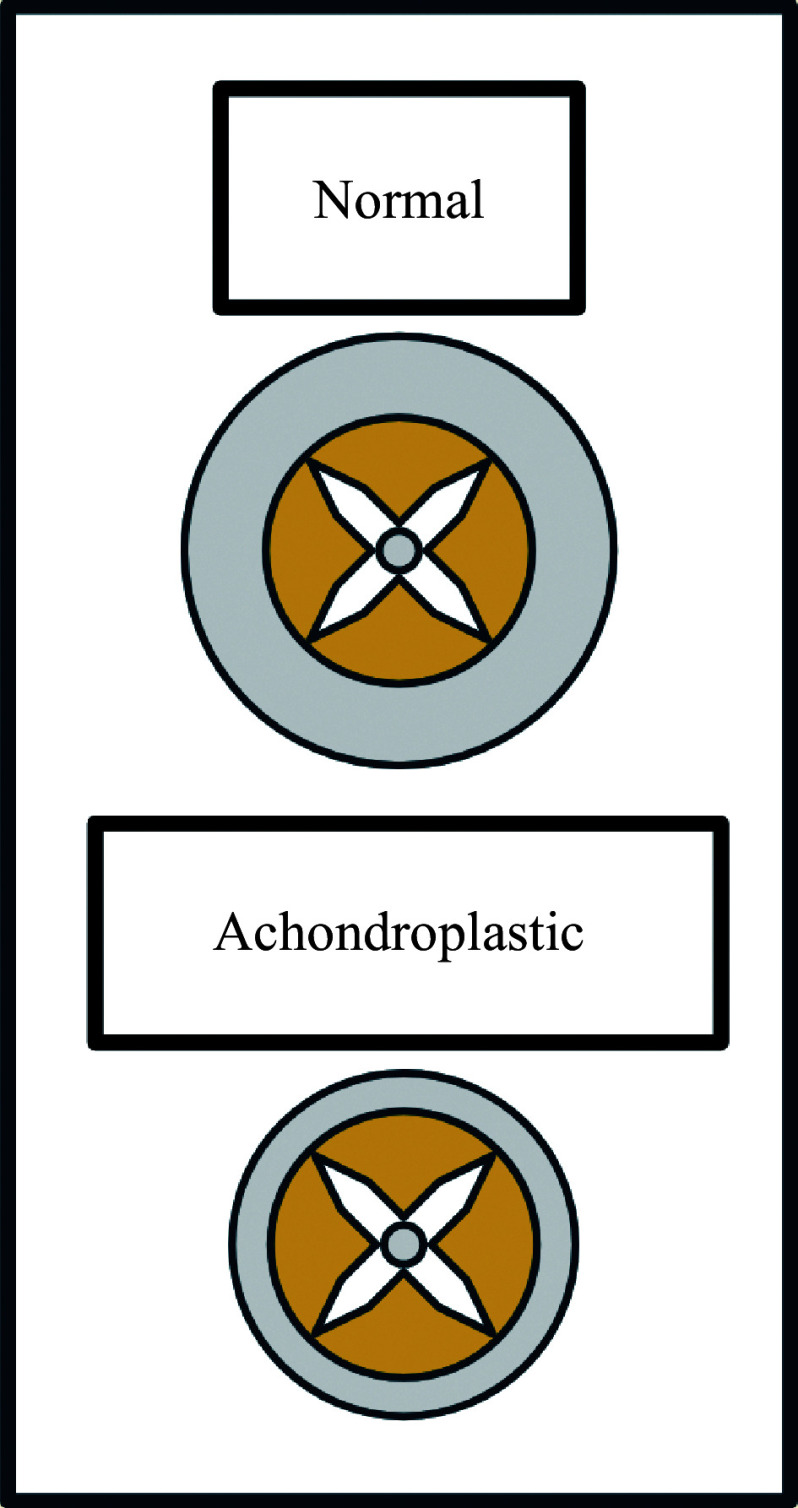
Schematic comparison of the spinal cord and canal between the normal and achondroplasia.

**Figure 2:**
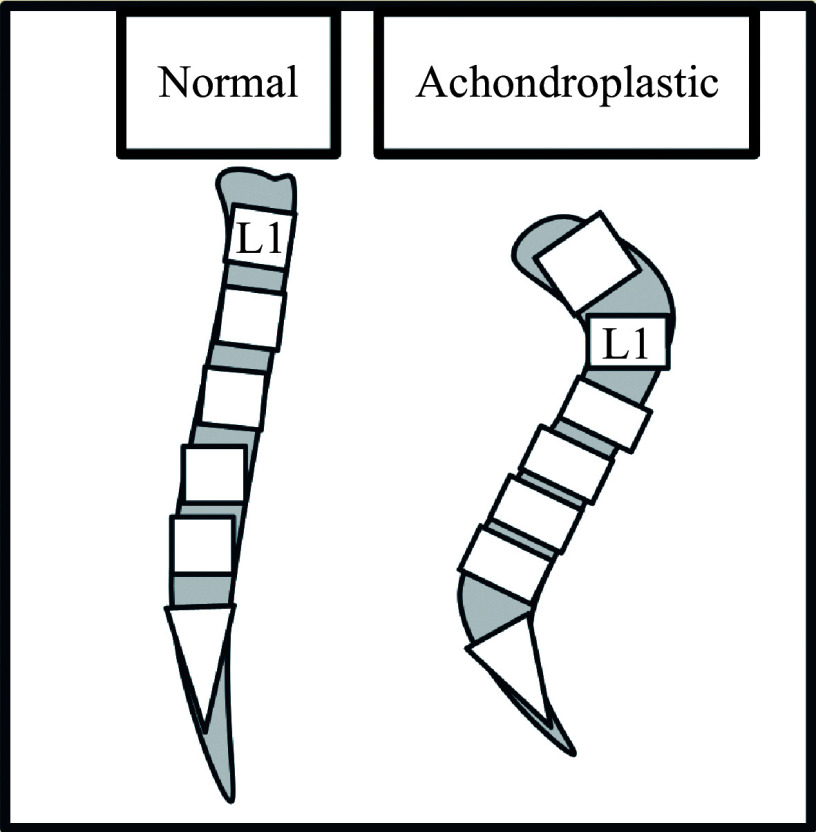
Schematic illustration of thoracolumbar kyphosis and lumbar lordosis commonly seen in achondroplasia, compared with normal.

Neuraxial techniques tend to be more challenging in achondroplastic patients. The spinal canal is narrower, but the spinal cord remains largely unchanged, causing it to occupy a larger proportion of space, compared with normal (***Fig. 1***). This leads to a tighter subarachnoid and epidural space, which, in addition to prominent intervertebral discs, may make the access more difficult. Dorso-lumbar kyphosis and lumbar lordosis may also affect the angle of the needle to reach the target location^[2,9]^ (***Fig. 2***). Though spine imaging may be beneficial for planning purposes, an ultrasound-guided technique may be used, especially in patients who have previously undergone lumbar decompressions with scar tissues present^[8]^. Using ultrasound during neuraxial techniques may help with landmark identification, reduce the number of attempts, determine skin-to-epidural distance, and estimate needle angle, though its use is limited by a lack of technical expertise^[10]^.

Other than the anatomy, the question remains: what the choice of an optimal dose for spinal medication in such patients is? The challenge lies in achieving an appropriate surgical level without the feared complication of a high neuraxial block. Achondroplasia causes disproportionate dwarfism that mainly affects the limbs rather than the axial skeleton; however, when combined with spinal stenosis, it creates confusion for dosing. Case reports have aggregated available data on successful spinal anesthesia used in achondroplastic patients, showing a range of 5 mg to 10 mg of hyperbaric bupivacaine used for C-sections in patients with heights ranging from 109 cm to 148 cm^[8,11]^. This highlights a large dose range with the only consistent feature being a dose reduction. Unfortunately, because of the high variability among these patients, there are no clear guidelines for the exact dose of local anesthetics in achondroplastic patients, other than a general recommendation for dose reduction.

With the single-shot spinal anesthetic dose and volume question being largely unanswered, a titratable neuraxial method may be more desirable. Both CSE and continuous spinal methods allow for such titration. An epidural alone would take a somewhat longer time to achieve an adequate surgical block, especially in an emergent situation. To date, there are no case reports of a continuous spinal being used in an achondroplastic patient, although theoretically, it would be possible^[12]^.

GA is risky in parturients, particularly because of airway management concerns, which arise from the edematous and more vascular airway, the decreased functional residual capacity, the increased oxygen requirements, the reduced lower esophageal sphincter tone, the increased gastric reflux, the delayed gastric emptying, and the enlarged breasts that complicate the insertion of the laryngoscope^[13]^. In obese patients, additional factors such as a large tongue, redundant oropharyngeal tissue, cervical and thoracic fat pads, high neck circumference, the decreased chest wall compliance, and the increased oxygen requirements may also contribute to the airway concerns and risks of GA^[14]^. The airway difficulties are compounded by unique characteristics of achondroplastic patients, including macrocephaly with a prominent forehead, macroglossia, cervical instability, midfacial retrusion, small mouth opening, short neck, rib hypoplasia, the decreased functional residual capacity, and restrictive lung disease^[15]^. Difficult intubation, mask ventilation, and cervical instability necessitate the consideration of advanced airway techniques, such as awake fiberoptic or awake Glidescope techniques^[16]^. There may be times that necessitate GA (patient preference, emergent nature, bleeding, clotting disorder, *etc.*); therefore, the challenges in airway management may increase the risk of failed intubation.

In conclusion, the present case showed that a successful obstetric outcome may be achieved in an achondroplastic patient using a titratable form of neuraxial anesthesia. Although there is no consensus on which regional technique is most appropriate for cesarean delivery in such a parturient, anatomical abnormalities, duration of the procedure, and variability in drug distribution play a crucial role in the choice of anesthetic technique. Electing a regional block over GA avoided the risk of potentially difficult intubation in a morbidly obese patient with challenging craniofacial features and increased patient satisfaction. However, this case highlights the importance of understanding the anatomical and physiological changes present in an achondroplastic pregnant woman, and patient care should be individualized.
